# Disaster-related deaths with alcohol-related diseases after the Fukushima Daiichi nuclear power plant accident: case series

**DOI:** 10.3389/fpubh.2023.1292776

**Published:** 2024-01-15

**Authors:** Kemmei Kitazawa, Toyoaki Sawano, Yuna Uchi, Moe Kawashima, Hiroki Yoshimura, Michio Murakami, Saori Nonaka, Hiroaki Saito, Mamoru Sakakibara, Kazuko Yagiuchi, Mako Otsuki, Akihiko Ozaki, Chika Yamamoto, Tianchen Zhao, Taiga Uchiyama, Tomoyoshi Oikawa, Shinichi Niwa, Masaharu Tsubokura

**Affiliations:** ^1^Department of Radiation Health Management, Fukushima Medical University, Fukushima, Japan; ^2^Department of Surgery, Jyoban Hospital of Tokiwa Foundation, Iwaki, Japan; ^3^Department of Health Risk Communication, Fukushima Medical University, Fukushima, Japan; ^4^Department of Internal Medicine, Soma Central Hospital, Fukushima, Japan; ^5^Reinstatement Support Center for Nurses, Incorporated Foundation of Tokiwa-kai, Iwaki, Japan; ^6^St. Olive Nursing Home, Shirakawa, Japan; ^7^Department of Nursing, Fukushima Medical University Hospital, Fukushima, Japan; ^8^Department of Breast and Thyroid Surgery, Jyoban Hospital of Tokiwa Foundation, Iwaki, Japan; ^9^Department of Thyroid and Endocrinology, Fukushima Medical University, Fukushima, Japan; ^10^Department of Neurosurgery, Minamisoma Municipal General Hospital, Minamisoma, Japan; ^11^Department of Psychiatry, Aizu Medical Center, Fukushima Medical University, Aizuwakamatsu, Japan

**Keywords:** disaster-related deaths, alcohol-related diseases, radiation disaster, indirect-effect, evacuation

## Abstract

**Introduction:**

The health of patients with mental disorders, such as alcohol-related diseases, often deteriorates after disasters. However, the causes of death among those with alcohol-related diseases during and after radiation disasters remain unclear.

**Methods:**

To minimize and prevent alcohol-related deaths in future radiation disasters, we analyzed and summarized six cases of alcohol-related deaths in Minamisoma City, a municipality near the Fukushima Daiichi nuclear power plant.

**Results:**

Patients were generally treated for alcohol-related diseases. In one case, the patient was forced to evacuate because of hospital closure, and his condition worsened as he was repeatedly admitted and discharged from the hospital. In another case, the patient’s depression worsened after he returned home because of increased medication and drinking for insomnia and loss of appetite.

**Discussion:**

The overall findings revealed that, in many cases, evacuation caused diseases to deteriorate in the chronic phase, which eventually resulted in death sometime after the disaster. To mitigate loss of life, alcohol-related diseases must be addressed during the chronic phases of future large-scale disasters, including nuclear disasters.

## 1 Introduction

Mental disorders are an important aspect of the indirect health effects after radiation disasters. Environmental changes influence mental disorders significantly (1). Post-disaster changes in the environment, such as the collapse of infrastructure, loss of social interaction with family and neighbors, and changes in living spaces due to evacuation, are particularly pronounced and occur simultaneously. Disasters often introduce risk factors that can worsen mental disorders. Previous studies have reported that post-traumatic stress disorder (PTSD), depression, schizophrenia, and suicidal ideation usually increase after radiation disasters (2–4). Additionally, stress reactions due to disasters can promote acute exacerbations of illnesses that were stable before disaster occurrence (1).

Alcohol-related diseases comprise a category of mental disorders that requires special attention during the disaster recovery period. These diseases include alcohol use disorder, liver cirrhosis, and pancreatitis. A previous report showed that the frequency of alcohol use by residents in disaster-affected areas increased after the disasters; examples of such individuals include survivors of the September 11, 2001 terrorist attack in the United States and accident reconstruction workers during the Chernobyl nuclear power plant accident (5). Alcohol-related disorders represent conditions that, without appropriate social and therapeutic interventions, may lead to fatal outcomes, including suicide or cirrhosis. The implementation of effective responses is essential, particularly during periods of disaster, to address these challenges comprehensively (6). Contrarily, knowledge is insufficient surrounding the post-disaster deaths of residents with alcohol-related diseases who were evacuated during radiation disasters.

Minamisoma City, Fukushima Prefecture, located near the Tokyo Electric Power Company (TEPCO) Fukushima Daiichi Nuclear Power Plant (FDNPP), was severely affected by the Great East Japan Earthquake and subsequent tsunami, as well as the FDNPP accident in March 2011 (triple disasters). Some areas of Minamisoma were set as evacuation zones, forcing residents to relocate. In addition to direct deaths from the earthquake and tsunami, disaster-related deaths associated with evacuation following the FDNPP accident constitute a major issue (7). As of March 31, 2022, a total of 520 people were certified as having indirectly died due to the disasters (in this paper, the term “disaster-related deaths” refers to deaths that are indirectly caused by a disaster) in Minamisoma City; this is the highest number among the municipalities in the six Tohoku prefectures, accounting for approximately 22.2% of the total number of disaster-related deaths in the Fukushima Prefecture (8, 9). Evidence suggests a connection between prolonged evacuations and the psychological impact associated with these disaster-related deaths (10). For instance, after the FDNPP accident, previous reports suggested that those who evacuated outside the Fukushima Prefecture had a risk of depression particularly with worries about dislocation and condolence money (2). Furthermore, evacuees with problems regarding neighbors in evacuated areas, harmful rumors, and family division and discord had an increased risk for PTSD (2, 11). Moreover, an elevated incidence of mental disorders is associated with alcohol. For instance, people who did not drink before the FDNPP accident were more likely to develop new drinking habits after the disaster, and post-disaster drinking habits caused psychological problems (12, 13). Out-of-prefecture evacuees experienced more psychological distress than in-prefecture evacuees (13). These complicated multifocal factors lead to increased social isolation and decreased social support (2). Although information on medical provisions among psychiatric hospitals in the area is very limited, existing literature reports that evacuations were properly conducted in psychiatric hospitals. Therefore, outpatient care may have been delayed for a certain period (14, 15). Alcohol-related disorders are characterized by being more susceptible to various environmental changes, such as stress and treatment interruptions, compared with other illnesses (16). To date, no information exists on individuals with alcohol-related diseases who were certified as disaster-related deaths after the FDNPP accident, and appropriate countermeasures have not been determined.

In this study, we extracted cases in which alcohol-related diseases were involved in the deaths from a dataset of all disaster-related deaths in Minamisoma City. We then analyzed and summarized those cases, focusing on the presence of alcohol-related diseases before the disaster, the number of days between the disaster and death, the number of evacuations, and the presence of stress. The cases of disaster-related deaths due to alcohol-related diseases in Minamisoma City must be analyzed to minimize and prevent disaster-related deaths due to alcohol-related diseases following future radiation disasters.

## 2 Case presentation

Data regarding the 520 individuals officially recognized as having disaster-related deaths in Minamisoma City were collected by obtaining information from the city office in response to applications from bereaved families. During the process of data collection, we could not access medical information from hospitals or any other information sources. We did not use other publicly available materials (17).

The average age at death of all the participants certified as disaster-related deaths in Minamisoma City was 82.69 years. Among these, six cases were certified as disaster-related deaths associated with alcohol-related diseases. These six patients were younger than the overall population, with an average age of 52.83 years. In addition, while the average number of days from disaster to death for all participants was 230.6 days, the six selected individuals had an average of 341.5 days, indicating a longer time until death.

In this report, alcohol-related diseases are caused by alcohol intake and refer to psychiatric diseases such as alcohol dependence and liver diseases, including alcoholic cirrhosis. The following codes were considered to represent alcohol-related diseases: F10, K70, G31, E244, E52, F04, F058, F060, G405, G621, G701, G721, H470, I426, K292, K852, K860, Q860, and R780, in ICD10 (Table 1).

**Table 1:** Summary of the six cases of disaster-related deaths associated with alcohol-related diseases.

No.	Direct cause of death	Sex	Age at death	Number of evacuations	Medical history	Stress (leading to death)	Days from disaster to death	Evacuation housing
1	Liver failure	Male	40	4	Cirrhosis, Esophageal varices	Yes	961 days	Relatives’ house, renting house
2	Multiple organ failure	Male	49	3	Diabetes mellitus, alcoholic cirrhosis, left humerus fracture, left elbow fracture, hepatic encephalopathy, hypertension, and hyperammonemia	No	265 days	Emergency temporary housing
3	Cirrhosis	Male	55	1	Diabetes, hypertension, alcoholic cirrhosis, and fractures of both ankle joints	Yes	27 days	Relative’s house
4	Cirrhosis of the liver (alcoholic)	Male	57	0	Cirrhosis of the liver (alcoholic)	Unknown	6 days	Hospital
5	Hepatic encephalopathy	Female	71	1	Diabetes, dementia, and cirrhosis (hepatic encephalopathy)	Unknown	72 days	Emergency temporary facility, Hospital
6	Suicide by hanging oneself	Male	45	2	Depression, gastric ulcer	Unknown	718 days	Unknown

## 3 Timeline of the cases and diagnostic assessment

1. A 40-year-old man with deteriorated liver function before the disaster was treated for alcohol use disorder. He temporarily returned home after the disaster; however, because of stress at work, he returned to his apartment in the evacuation area and changed jobs. During this process, the patient often fell ill. He had a habit of secretly drinking alcohol while publicly claiming to refrain from drinking. He experienced great stress due to his wife’s miscarriage, another pregnancy, changing jobs, and the death of his mother-in-law. Thereafter, he was diagnosed with liver cirrhosis and esophageal varices. Approximately 2 years and 8 months after the disaster, the patient died of liver failure.
2. A 49-year-old man was hospitalized for diabetes and cirrhosis of the liver during the disaster. He had been recuperating at home because of hospital closure and was forced to evacuate. He was admitted and discharged from the hospital repeatedly because of his worsening health conditions. His liver dysfunction worsened, and during the outpatient treatment period, he fell at his home and was hospitalized for a fracture. Approximately 8 months after the disaster, he died of multiple organ failure.
3. A 55-year-old man with a medical history of diabetes, hypertension, and alcoholic cirrhosis was unable to evacuate until March 21. Until then, he stayed at his home where the infrastructure was disrupted. Because he was scheduled to visit a doctor on March 12 for a regular examination, he was not on medication. He retired 1 year before the disaster and could walk only slowly after breaking both ankle joints. He spent time doing housework, tending to plants and flowers. After the disaster, the evacuation prompted him to spent more time inside his home watching television. He experienced a combination of stress from the flooding of his home, long travel hours, and changes in his environment after the evacuation. Twenty-seven days after the earthquake, he died of cirrhosis.
4. A 57-year-old man with alcoholic cirrhosis was hospitalized because of liver cirrhosis at the time of the disaster. However, the hospital was unable to provide adequate medical treatment because of the confusion regarding the disaster. Six days after the earthquake, the patient died in the hospital without evacuation.
5. A 71-year-old woman with diabetes, dementia, and alcoholic cirrhosis required assistance with all daily activities. She was hospitalized 1 month before the disaster because of alcoholic liver disorders. She was suddenly discharged from the hospital after the disaster, and her brother’s wife took care of her. She died of hepatic encephalopathy within 3 months of the disaster.
6. A 45-year-old man with a history of depression and gastric ulcer was evacuated from the Fukushima Prefecture and returned to his home in Minamisoma City. Thereafter, he experienced insomnia and loss of appetite and began taking mood stabilizers. He was treated at a psychosomatic clinic, but his drug abuse and alcohol consumption increased, and he died of suicide approximately 2 years after the disaster.

## 4 Discussion

After the FDNPP accident, five of the six patients who were certified as disaster-related deaths due to alcohol-related diseases in Minamisoma City, an area severely affected by disaster in the long term, had been treated for alcohol-related diseases from the pre-disaster period. The treatment regimens of all six were affected in some way by the evacuation orders resulting from the accident. The six patients were divided into two groups: those who died in the acute phase (3, 4, and 5) and those who died in the chronic phase (1, 2, and 6) after the disaster. The other divisions are 1, 6, 2, 3, 4, and 5, but they are for reference only. In cases 1 and 6, the contribution of psychological problems to the death was so great that a psychiatrist should have been involved. In cases 2–5, the first problem was alcohol, but the physical health was already very weak when the disaster occurred, and the post-disaster confusion did not compensate for that weakness.

In the acute phase of the FDNPP accident, disaster-related deaths associated with alcohol-related diseases may have resulted from the disruption of medical care and management of patients with alcohol-related diseases who required continuous treatment. Patient 4, who was not evacuated, reportedly could not receive adequate treatment because normal hospital functions were disrupted in the disaster-affected areas, although the detailed treatment process from March 11 to March 16 was not revealed. In addition to the stress caused by the evacuation, earthquakes, tsunamis, and concerns about radiation exposure made normal function impossible for the hospitals in the affected areas. Consequently, hospitals lacked the necessary drugs, nurses, and other personnel to treat alcohol-related diseases, and this inability to provide adequate treatment could have been a contributing factor to disaster-related deaths. In Minamisoma City, medical personnel such as nurses were also evacuated, resulting in a shortage of staff and supplies necessary for care, such as medicine and oxygen (18, 19). A similar situation occurred during Hurricane Katrina in the United States in 2005; many staff members in nursing homes (NHs) worked while worrying about their families and homes, while other NH personnel evacuated with their own families, leading to inadequate staffing (20, 21). The shortage of human resources and supplies is not limited to Minamisoma City after the FDNPP accident, and this shortage may be associated with disaster-related deaths.

Even those who did not have alcohol-related diseases before the disaster may have increased their alcohol consumption in the wake of the nuclear power plant accident, potentially contributing to the related suicides. This was exactly the case for patient 6. The patient had a history of depression before the accident, and insomnia and loss of appetite were observed immediately after the accident. He received psychological treatment, but his alcohol consumption increased, and he eventually committed suicide. This case study suggests that some cases of depression diagnosed after the nuclear power plant accident may have been overlooked, even though the patients may have suffered from alcohol-related diseases due to the increased frequency of alcohol consumption.

Patients with alcohol-related diseases in the chronic phase of a disaster are likely to suffer from negative health effects and death due to changes in the hospitals and environments in which they receive treatment following evacuation. In many cases, disasters cause hospitals to close, and patients must be prematurely discharged, as in the case of patient 2. Four of the six patients were evacuated from the Fukushima Prefecture immediately after the accident. Evacuation could induce stress owing to changes in workplaces and the stress of living in an unfamiliar place. In addition to these stressors, patient 1 also experienced stress from his wife’s miscarriage, another pregnancy, a new job, and the death of his mother-in-law. Patient 3 retired 1 year before the earthquake. Before the disaster, he used to do housework and tend to his plants and flowers during the daytime, but the evacuation prompted him to spend more time inside his home watching television. Chronic diseases such as diabetes mellitus and dyslipidemia have been reported to worsen as a result of spending most of the time indoors instead of being active outside (22).

As mentioned above, two distinct scenarios are evident in the acute and chronic phases. In the acute phase, individuals could not receive adequate treatment due to shortages in medication and personnel, leading to fatalities. In the chronic phase, the hospital where they were receiving outpatient care was closed due to the disaster, causing a disruption in treatment. The additional factors related to evacuation introduce various environmental changes and stress, which may exacerbate illness and result in death. Considering the chronic nature of alcohol-related diseases, over time following the disaster, some bereaved families of patients with alcohol-related diseases may not have realized that increases in alcohol consumption were related to the disaster and therefore may not have applied to their municipal office for the distinction of disaster-related death. Although only the six cases discussed here were certified as disaster-related deaths in Minamisoma City, more people may have died from alcohol-related diseases owing to the indirect effects of radiation disasters.

Given the characteristics of patients with alcohol-related diseases, for whom ongoing treatment is essential, resources for their treatment and management should be devoted to the chronic phase of radiation disasters, in addition to the acute phase. In all of the cases discussed in this report, the patients’ lifestyles changed after the nuclear power plant accident, causing increased stress and strain on their bodies. Drinking behavior is known to be used as a convenient means of stress coping to “relieve fatigue,” “change my mood,” “sleep well,” and “like the atmosphere of the place where I drink” according to the paper by Shirakura (23). Patients 1 and 2 were hospitalized for treatment of alcoholic-related diseases but were forced to temporarily suspend treatment due to the evacuation, after which they received treatment at home. In addition to being accustomed to drinking alcohol at home, they resumed this behavior while under the stress of changes in their living environments. The relationship between alcohol dependence and post-disaster evacuation was not limited to the aftermath of the Fukushima accident. Six months after the 1995 Great Hanshin-Awaji Earthquake, symptoms of alcohol dependence became noticeable in evacuation areas, and 12 months later, some cases of lonely deaths were reported in temporary shelters, likely related to alcohol dependence (24). In the long-term aftermath of disasters, including nuclear disasters and other major catastrophes, the treatment of alcohol-related diseases is crucial.

Intervention can be effectively applied in two critical domains, ensuring the continuity of treatment both before and after disasters and mitigating the stress induced by relocation and environmental changes during evacuation. Facilitating access to the patient’s medical information is imperative, even at the evacuation destination, and a seamless handover and prompt resumption of treatment at the evacuation site are paramount. Prolonged lapses in treatment are associated with a heightened risk of relapse. In the treatment of patients with alcohol-related disorders, the post-disaster environment involves substantial changes, rendering individuals more susceptible to stress. Healthcare providers must be particularly vigilant when attending to the information shared by patients. Given the observed instances of increased alcohol consumption in such situations, the need for targeted psychological support post-disaster is elevated, compared with that during the pre-disaster period. Alcohol-related diseases in the chronic phase of disaster frequently stem from stress triggered by environmental changes, and this stress can be mitigated through careful medical attention and other interventions, effectively averting the worsening of the condition (16). Our study provided detailed descriptions of chronic cases to highlight the significance of this relationship.

This study had several limitations. First, this case series utilizes data analysis based on aggregated information from individuals certified as disaster-related deaths in Minamisoma City. Consequently, no additional information was available for each case beyond that which was documented in this dataset. Hence, note the limited sample size due to the specificity of this data in question and discussion may remain insufficient surrounding the ways through which alcohol-related diseases following disasters lead to death and at which point the events become fatal. Second, prior studies have suggested that the degree of exposure to a natural disaster alone may not be correlated with changes in drinking behavior in the aftermath and that possible changes in drinking behavior may be more dependent on factors secondary to the trauma itself, such as economic loss, relocation, and disruption of normal life (25), the interpretation of this study should be considered with great care. Third, some literature has suggested that the extensively documented association between disaster exposure and changes in alcohol consumption might be overestimated, so drinking behavior in the aftermath of disasters may not necessarily associated with the disaster itself (25–27).

## 5 Conclusion

According to the dataset of the disaster-related deaths in a single municipality near the FDNPP after the nuclear disaster, six of the disaster-related deaths were attributed to alcohol-related diseases. Many deaths occurred after the evacuation of residents from their homes, as the diseases worsened during the chronic phase. Considering the large number of deaths that occurred after the disaster, some cases of death owing to alcohol-related diseases might have been overlooked. Patients undergoing treatment for alcohol-related diseases, as well as those who have a tendency to increase their alcohol consumption with stress, require careful monitoring and appropriate medical attention at evacuation centers and other healthcare facilities following disaster. Management of alcohol-related diseases is important to prevent disaster-related deaths, and patient support is necessary for future large-scale disasters, including nuclear disasters, especially in the chronic phase.

## Data availability statement

The raw data supporting the conclusions of this article will be made available by the authors, without undue reservation.

## Ethics statement

This retrospective study was approved by the Ethics Committee of Minamisoma Municipal General Hospital (approval number: 2-21) and Fukushima Medical University (approval number: 2020-297). The study was conducted using an existing administrative database of disaster-related deaths provided by the municipality. Information regarding the study was publicly disclosed using an opt-out approach in accordance with institutional guidelines. Written informed consent from the patients/participants legal guardian/next of kin was not required to participate in this study in accordance with the national legislation and the institutional requirements because the study used anonymized data collected for administrative purposes. The study was conducted in accordance with the principles of the Declaration of Helsinki.

## Author contributions

KK: Writing – original draft, Writing – review & editing. TS: Writing – original draft, Writing – review & editing. YU: Data curation, Formal analysis, Writing – review & editing. MK: Data curation, Formal analysis, Writing – review & editing. HY: Writing – review & editing. MM: Writing – review & editing. SaN: Writing – review & editing. HS: Writing – review & editing. MS: Data curation, Writing – review & editing. KY: Data curation, Writing – review & editing. MO: Data curation, Writing – review & editing. AO: Writing – review & editing. CY: Writing – review & editing. TZ: Data curation, Formal analysis, Writing – review & editing. TU: Writing – review & editing. TO: Data curation, Writing – review & editing. ShN: Conceptualization, Writing – review & editing. MT: Conceptualization, Data curation, Writing – review & editing.
