# Correction: Disaster-related deaths with alcohol-related diseases after the Fukushima Daiichi nuclear power plant accident: case series

**DOI:** 10.3389/fpubh.2026.1834927

**Published:** 2026-05-14

**Authors:** Kemmei Kitazawa, Toyoaki Sawano, Yuna Uchi, Moe Kawashima, Hiroki Yoshimura, Michio Murakami, Saori Nonaka, Hiroaki Saito, Mamoru Sakakibara, Kazuko Yagiuchi, Mako Otsuki, Akihiko Ozaki, Chika Yamamoto, Tianchen Zhao, Taiga Uchiyama, Tomoyoshi Oikawa, Shinichi Niwa, Masaharu Tsubokura

**Affiliations:** 1 Department of Radiation Health Management, Fukushima Medical University, Fukushima, Japan; 2 Department of Surgery, Jyoban Hospital of Tokiwa Foundation, Iwaki, Japan; 3 Department of Health Risk Communication, Fukushima Medical University, Fukushima, Japan; 4 Department of Internal Medicine, Soma Central Hospital, Fukushima, Japan; 5 Reinstatement Support Center for Nurses, Incorporated Foundation of Tokiwa-kai, Iwaki, Japan; 6 St. Olive Nursing Home, Shirakawa, Japan; 7 Department of Nursing, Fukushima Medical University Hospital, Fukushima, Japan; 8 Department of Breast and Thyroid Surgery, Jyoban Hospital of Tokiwa Foundation, Iwaki, Japan; 9 Department of Thyroid and Endocrinology, Fukushima Medical University, Fukushima, Japan; 10 Department of Neurosurgery, Minamisoma Municipal General Hospital, Minamisoma, Japan; 11 Department of Psychiatry, Aizu Medical Center, Fukushima Medical University, Aizuwakamatsu, Japan

**Keywords:** disaster-related deaths, alcohol-related diseases, radiation disaster, indirect-effect, evacuation

The **Ethics statement** was erroneously given as

“The studies involving humans were approved by the Minamisoma Municipal General Hospital Ethics Committee (approval number: 2–21) and the Fukushima Medical University Ethics Committee (reference number: 2020-297). The studies were conducted in accordance with the local legislation and institutional requirements. Written informed consent for participation in this study was provided by the participants' legal guardians/next of kin.”

The correct **Ethics statement** is “This retrospective study was approved by the Ethics Committee of Minamisoma Municipal General Hospital (approval number: 2-21) and Fukushima Medical University (approval number: 2020-297). The study was conducted using an existing administrative database of disaster-related deaths provided by the municipality. Information regarding the study was publicly disclosed using an opt-out approach in accordance with institutional guidelines. Written informed consent from the patients/participants legal guardian/next of kin was not required to participate in this study in accordance with the national legislation and the institutional requirements because the study used anonymized data collected for administrative purposes. The study was conducted in accordance with the principles of the Declaration of Helsinki.”

The original version of this article has been updated.

